# A case report of piecemeal submucosal tunnel endoscopic resection for a giant esophageal leiomyoma larger than 8 cm

**DOI:** 10.1055/a-2526-2387

**Published:** 2025-03-12

**Authors:** Ping Ma, Limei Gu, Tingsheng Ling

**Affiliations:** 1 The First Clinical Medical College of Nanjing University of Chinese Medicine, Nanjing, China; 2 Department of Gastrointestinal Endoscopy, Affiliated Hospital of Nanjing University of Chinese Medicine, Nanjing, China

A 26-year-old man presented with substernal discomfort. Enhanced computed tomography scans revealed a significant submucosal tumor with dimensions of 8.19×4.55×2.69 cm ( Fig. 1 **a** ). Endoscopy revealed a huge tumor on the posterior wall of the esophagus, protruding into the lumen Fig. 1 **b** ). Three-dimensional reconstruction demonstrated that the lesion was situated on the posterior wall of the esophagus, in proximity to the thoracic aorta ( Fig. 1 **c** ). After a thorough discussion regarding the risks and benefits, piecemeal submucosal tunnel endoscopic resection (P-STER) was performed to remove the tumor ( Video 1 ).

**Fig. 1:**
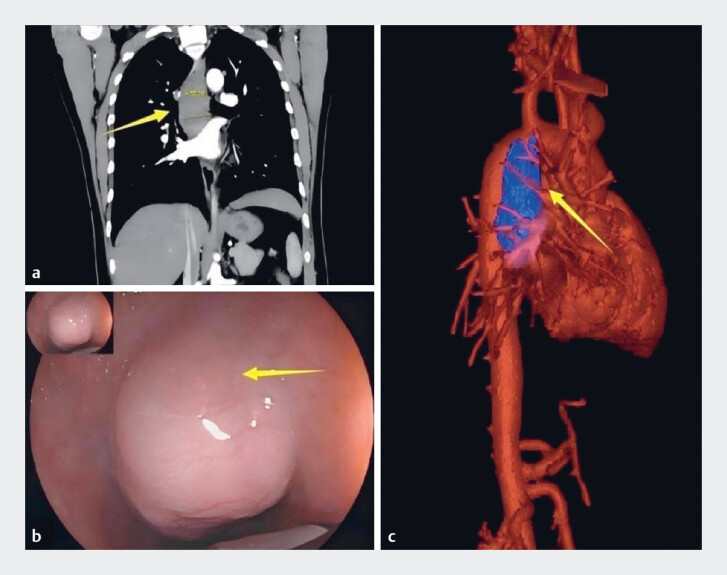
Computed tomography (CT) and endoscopy images of the lesion (arrow). **a** Enhanced CT scans revealed a significant submucosal tumor with dimensions of 8.19 × 4.55 × 2.69 cm. **b** Endoscopy revealed a huge tumor on the posterior wall of the esophagus, protruding into the lumen. **c** Three-dimensional reconstruction demonstrated that the lesion was situated on the posterior wall of the esophagus, in proximity to the thoracic aorta.

Piecemeal submucosal tunnel endoscopic resection for a giant esophageal leiomyoma larger than 8 cm.Video 1

A mucosal incision was made 5 cm above the lesion to establish a broad submucosal tunnel ( Fig. 2 **a** ). Dissection was performed within this tunnel along the tumor margins to free the submucosal and muscularis propria layers ( Fig. 2 **b** ). We attempted other methods without success. Finally, a high-frequency incision knife in conjunction with a snare was used to segment and excise the tumor ( Fig. 2 **c** ). Once hemostasis was achieved, the tunnel entrance was closed ( Fig. 2 **d** ). The patient resumed a liquid diet 4 days post-procedure and was discharged from hospital after a total of 15 days. Histopathological and immunohistochemical analyses confirmed a diagnosis of leiomyoma ( Fig. 3 ).

**Fig. 2:**
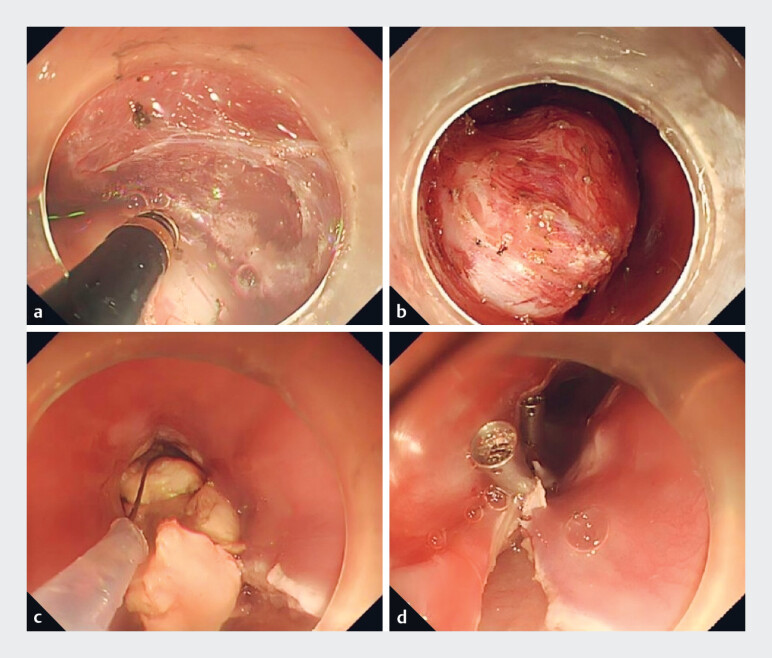
Surgical procedure images. **a** A submucosal tunnel was established. **b** Dissection was performed along the tumor margins to free the submucosal and muscularis propria layers. **c** The tumor was segmented and removed. **d** Titanium clips were applied to close the tunnel entrances securely.

**Fig. 3:**
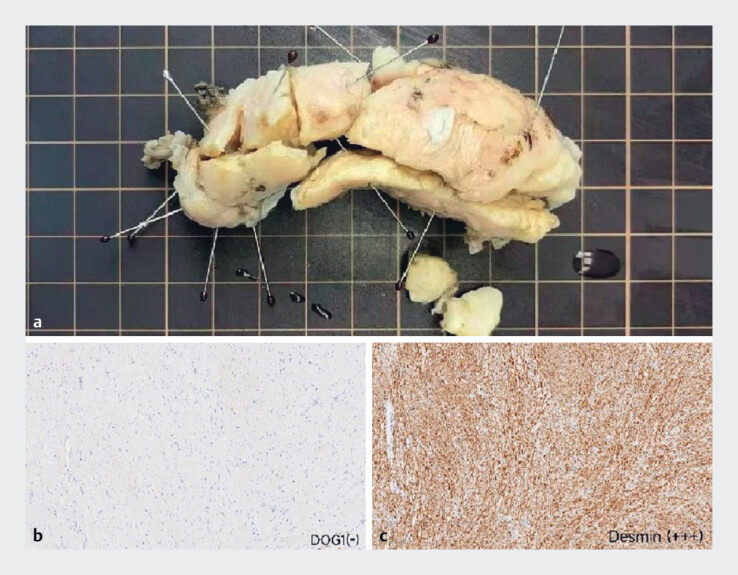
Histological evaluation. **a** The specimen was fixed outside the body, and measured approximately 9×6×3 cm. **b** Immunohistochemistry indicated DOG(–). **c** Immunohistochemistry indicated desmin (+++).

P-STER, a variant of the submucosal tunnel endoscopic resection (STER) technique, is commonly used for treating submucosal tumors. In this case, P-STER has proven successful for treating esophageal submucosal tumors exceeding 8 cm in size, offering both safety and efficacy. European guidelines recommend a maximum size of 35 mm for endoscopic en bloc resection 1 . We have shown that P-STER is feasible for esophageal leiomyomas larger than 8 cm, even though such cases exceed the size limits recommended by expert consensus. In our experience, P-STER broadens the applicability of STER surgery, effectively minimizing trauma and surgical risk 2 3 . Further studies are needed to evaluate the clinical value of this approach.

Endoscopy_UCTN_Code_TTT_1AO_2AG_3AZ

## Notes

Endoscopy E-Videos https://eref.thieme.de/e-videos
*E-Videos* is an open access online section of the journal *Endoscopy* , reporting on interesting cases and new techniques in gastroenterological endoscopy. All papers include a high-quality video and are published with a Creative Commons CC-BY license. Endoscopy E-Videos qualify for HINARI discounts and waivers and eligibility is automatically checked during the submission process. We grant 100% waivers to articles whose corresponding authors are based in Group A countries and 50% waivers to those who are based in Group B countries as classified by Research4Life (see: https://www.research4life.org/access/eligibility/ ). This section has its own submission website at https://mc.manuscriptcentral.com/e-videos .
